# Measurement Performance Improvement of Buried Strain Sensors for Asphalt Pavement Using Mesoscale Finite Element Simulation

**DOI:** 10.3390/s25123754

**Published:** 2025-06-16

**Authors:** Haiyang Hu, Gang He, Man Huang, Dongdong Han, Hongzhou Zhu, Yongli Zhao

**Affiliations:** 1 Guizhou Express Qian Tong Construction Engineering Co., Ltd., Guiyang 550001, China18684155700@163.com (M.H.); 2 National & Local Joint Engineering Laboratory of Transportation and Civil Engineering Materials, Chongqing Jiaotong University, Chongqing 400074, China; zhuhongzhouchina@cqjtu.edu.cn; 3 School of Transportation, Southeast University, Nanjing 210096, China; yonglizhao2016@126.com

**Keywords:** asphalt pavement, buried strain sensor, deformation compatibility, measurement stability

## Abstract

Accurately measuring strain in asphalt pavements using buried strain sensors remains challenging due to the temperature sensitivity and heterogeneity of asphalt mixtures. This study focuses on improving the measurement performance of buried strain sensors in asphalt mixtures through finite element simulations. First, the sensing errors of existing buried strain sensors in asphalt mixtures were analyzed based on laboratory experiments. Subsequently, the factors affecting the deformation compatibility between the sensor and the asphalt mixture were investigated, and the effect of asphalt mixture heterogeneity on the stability of the sensor measurements are discussed. More importantly, a series of optimization strategies for buried strain sensors are proposed. The findings suggest that the equivalent modulus of the buried strain sensor should closely match that of the asphalt mixture, and its encapsulation must avoid inducing any reinforcement effects. Considering the dynamic modulus range of the asphalt mixture, it is recommended to adopt the lower bound, such as 0.25 GPa, as the equivalent modulus of the buried sensor. To eliminate the stiffening effect, the encapsulation may utilize low-modulus flexible materials. The inherent heterogeneity of asphalt mixtures influences the measurement stability of buried strain sensors: a higher overall modulus leads to a more uniform internal strain distribution, whereas a larger nominal maximum aggregate size (NMAS) results in poorer strain field uniformity. Increasing the gauge length of the buried strain sensor to at least three times the NMAS significantly enhances measurement stability. This study provides valuable guidance for the design of buried strain sensors in asphalt pavement applications.

## 1. Introduction

Monitoring the internal mechanical responses of asphalt pavement is essential for optimizing pavement design, maintenance strategies, and accurately predicting service life [1,2,3]. Embedded sensing technology enables in situ monitoring of the mechanical behavior of asphalt pavements under actual traffic loading and environmental conditions. Currently, numerous research institutions have initiated experimental programs employing buried strain sensors to investigate pavement mechanical responses [4,5]. For example, large-scale sensor deployments have been implemented on test sections such as the NCAT and MnROAD test tracks in the United States and the RIOHTRACK test road in China. These in situ monitoring efforts provide valuable support for improving the mechanistic-empirical pavement design methods used in various countries [6,7,8].

It is generally accepted that buried strain sensors provide accurate, direct strain readings without the need for calibration [9,10]. However, these sensors may exhibit significant measurement errors when used in asphalt mixtures. For example, Tan et al. [11] evaluated the measurement performance of two buried strain sensors used in asphalt mixtures in static loading tests and showed that their relative errors were as high as 40% and 88%, respectively. Zhao et al. [12] investigated the ability of buried strain sensors to capture the internal dynamic strain of asphalt mixtures. Their results showed that although the sensor output waveform closely matched the shape of the actual deformation, its amplitude was only about 50% of the true value. Han et al. [13] conducted numerical simulations and found that as the temperature increases, the sensor output progressively underestimates the actual strain within the asphalt mixture. Li et al. [14] further confirmed the measurement errors induced by temperature in buried strain sensors through uniaxial loading tests, demonstrating that as the temperature increases from −10 °C to 50 °C, the relative measurement error rises from 10% to 50%.

To enhance the measurement performance of buried strain sensors in asphalt mixtures, various improvement strategies have been proposed. For example, secondary calibration through laboratory testing has been employed to compensate for measurement errors caused by the deformation mismatch between the sensor and the asphalt mixture [15,16]. In addition, the configuration and materials of buried strain sensors have been optimized to improve strain transfer efficiency and measurement accuracy [17]. However, the core challenge in accurately measuring internal strain in asphalt mixtures lies in their pronounced temperature sensitivity [18,19]. The modulus of asphalt mixtures exhibits a marked decline under high-temperature conditions and increases noticeably at lower temperature [20,21]. Buried strain sensors are typically produced of stable materials with a constant modulus. This mismatch results in deformation incompatibility between the sensor and the asphalt mixture, ultimately leading to inaccurate strain measurements. In addition, asphalt mixtures are typical composite materials [22]. The internal strain distribution within asphalt mixtures is non-uniform, with the deformation of aggregate-reinforcing components being significantly lower than that of the binder phase [23,24,25]. This non-uniform strain field poses a substantial challenge to the stability of measurements obtained from buried strain sensors.

The finite element method has emerged as a powerful tool for modeling mechanical interactions between buried strain sensors and asphalt mixtures. It allows efficient parametric analysis of the sensor geometry, material properties, and boundary conditions without the high cost or logistical constraints associated with physical experimentation [26,27,28]. Han et al. [29] simulated the impact of the interface between buried strain sensors and asphalt mixtures on measurement errors, and proposed an encapsulation design in which a high-modulus core is wrapped with a flexible layer. Similarly, Liu et al. [30] analyzed the secondary calibration coefficients of buried strain sensors in asphalt mixtures at different temperatures through finite element simulations, thereby improving the sensing performance of the sensors. Wang et al. [31] studied the impact of buried sensors on the structural mechanical properties of asphalt pavement and the structural optimization of sensor components. Moreover, finite element methods are widely applied in the simulation of pavement materials and structural mechanics, including rutting, fatigue, crack propagation, and dynamic load responses at both macroscopic and mesoscopic scales [32,33,34]. Therefore, the finite element analysis can provide an effective means to investigate the interaction between buried strain sensors and asphalt mixtures, as well as to optimize and improve the measurement performance of the sensors.

## 2. Objective

This study aims to enhance the measurement performance of buried strain sensors in asphalt mixtures through mesoscale finite element simulations. Initially, sensing errors associated with existing buried strain sensors were analyzed based on laboratory experiments. Key sensor components were then modeled, and the coarse aggregate skeleton of the asphalt mixture was reconstructed using rigid body dynamics, resulting in a heterogeneous asphalt mixture model incorporating the embedded sensor. The study further investigated the factors influencing deformation compatibility between the sensor and the surrounding mixture, and analyzed the impact of asphalt mixture heterogeneity on measurement stability. Importantly, a series of optimization strategies were proposed to improve sensor performance. The findings provide valuable guidance for the optimized design of buried strain sensors tailored to asphalt pavement applications.

## 3. Measurement Accuracy of Buried Strain Sensor

**Figure 1:**
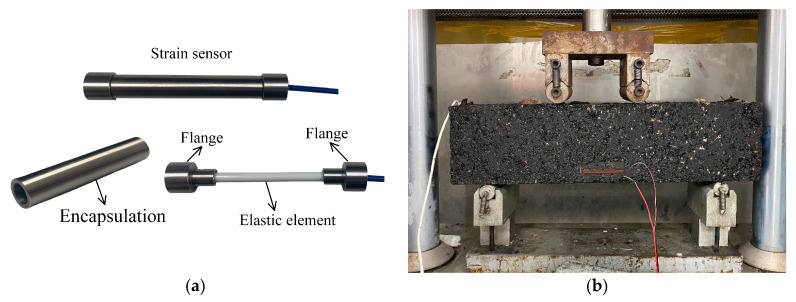
Evaluation of the sensing accuracy of buried strain sensor by four-point bending beam test: (**a**) buried strain sensor, (**b**) four-point bending beam tests.

As shown in Figure 1a, the buried strain sensor used in the experimental tests is an AS-200 model based on fiber Bragg grating technology, manufactured by a Chinese supplier. The internal structure of the AS-200 sensor mainly comprises two end flanges, a central elastic strain beam and an encapsulation. This sensor offers a measurement resolution of up to 1 με. Since the bending state at the bottom of the beam is similar to the bending behavior of the asphalt layer in real pavement structures, a four-point bending beam test was chosen to evaluate the measurement accuracy of the embedded strain transducer. The four-point bending beam tests were conducted with reference to the test method T0851-2009 in the Chinese specification [35]. Although this standard is not specifically designed for asphalt mixtures, it provides a test configuration that enables the generation of a pure tensile region in the specimen, which is suitable for our experimental purpose. Figure 1b illustrates the principle of the laboratory test. The asphalt mixture beam measures 400 mm × 100 mm × 100 mm. The buried strain sensor was installed at the mid-span, positioned 20 mm from the bottom surface, with its sensing direction aligned with the longitudinal axis of the beam. A resistive strain gauge was attached to the side surface of the beam at the same height as the buried strain sensor to provide a reference measurement of actual strain. The beam was upheld by two clamps spaced 300 mm apart and the upper surface was subjected to a vertical load with a span of 100 mm. The vertical load was applied under monotonic displacement control at a rate of 50 mm/min. The load cell used has a maximum capacity of 25 kN, which was sufficient for the scope of this experiment. The asphalt mixture used was AC-20, with a NMAS of 19 mm and an asphalt content of 5.1%. The tests were conducted at temperatures of 0 °C, 15 °C, 30 °C, and 40 °C.

**Figure 2:**
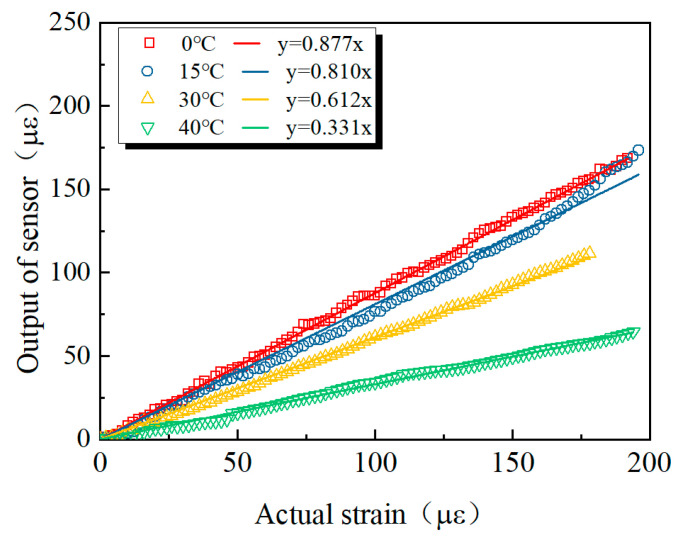
Measurement results of the buried strain sensor.

Figure 2 shows the comparison between the strain measured by the buried strain sensor and the actual strain under all temperature conditions. A clear linear relationship exists between the two, with the fitting performed using a line passing through the origin. However, the slope of the fitted line is consistently less than 1, indicating that the buried strain sensor systematically underestimates the actual deformation. Notably, the phenomena between the measured strain and the actual strain becomes more pronounced as the temperature increases. The observed differences may be attributed to the contrasting thermal behaviors of the two materials: the modulus of the asphalt mixture decreases with increasing temperature [36,37], while that of the buried strain sensor remains nearly constant. Consequently, as temperature rises, the ratio of the sensor modulus to that of the asphalt mixture increases, resulting in relatively smaller deformation of the sensor and thus a progressive underestimation of the measured strain.

## 4. Development of Mesoscale Finite Element Model

### 4.1. Geometry

**Figure 3:**
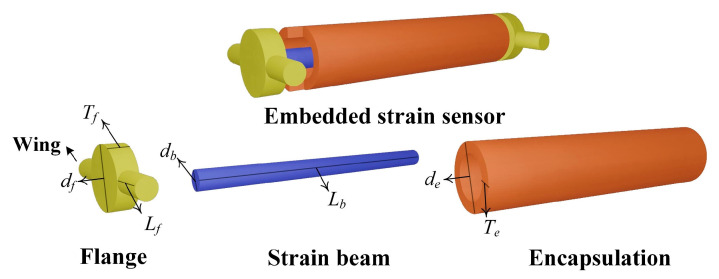
Schematic structure of buried strain sensor.

Figure 3 illustrates the structural schematic of the buried strain sensor. A typical sensing element consists of two flanges and a central elastic strain beam. To prevent interfacial debonding between the flanges and the surrounding asphalt mixture under cyclic loading, two lateral wings are integrated on both sides of the flanges along the vertical measurement direction. These wings enhance the mechanical bonding between the flanges and the asphalt matrix. However, the addition of flanges may lead to stress concentration, particularly when the strain sensor exhibits poor mechanical compatibility with the deformation of the asphalt mixture. The relative displacement between the two flanges induces tensile or compressive deformation in the elastic strain beam. Transition elements, such as fiber Bragg gratings or resistive strain gauges, are bonded to the elastic strain beam to monitor its deformation. As a result, the buried strain sensor can effectively measure the average strain within the asphalt mixture. To ensure long-term durability under traffic loads and adverse environmental conditions, the sensing element and the transition element are enclosed within a protective housing.

**Figure 4:**
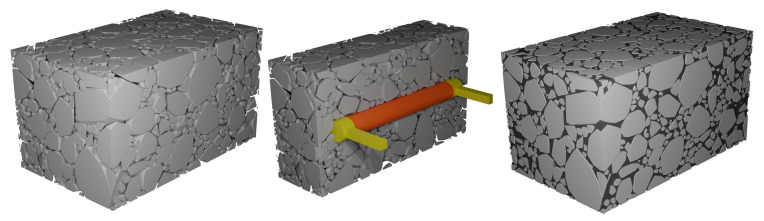
Mesoscale reconstruction of asphalt mixtures with a buried strain sensor.

To investigate the influence of asphalt mixture heterogeneity on the measurement performance of buried strain sensors, this study employed a mesoscale structural reconstruction method previously developed by the authors [38]. This method is based on rigid body dynamics to simulate the motion and interaction of aggregate particles. A digital aggregate library was first established by performing three-dimensional scans of actual aggregate particles. By using the digital aggregates in the library together with a rigid-body dynamics algorithm, a stable aggregate skeleton of the asphalt mixture can be generated. This method enables a one-to-one reconstruction of the mesoscale structure of asphalt mixtures. When incorporating the buried strain sensor model, the sensor geometry was predefined at a designated location to avoid spatial overlap with the aggregates. Considering computational limitations, the asphalt mixture specimen used in this study is set to dimensions of 14 cm × 8 cm × 8 cm. Figure 4 illustrates the heterogeneous structure of the asphalt mixture with a buried strain sensor. The sensor dimensions in this study are based on the AS-200 model, with a diameter of 10 mm, a flange length of 10 mm, and a total length of 80 mm. The gauge length of the sensor is defined by the flange spacing, resulting in a gauge length of 60 mm.

### 4.2. Mesh

**Figure 5:**
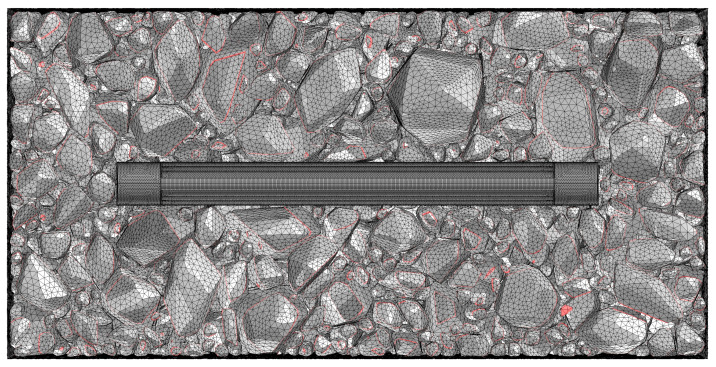
Mesh for heterogeneous geometry.

As shown in Figure 5, tetrahedral elements were employed to capture the heterogeneous geometry. Mesh sensitivity analysis indicated that element sizes should be limited to 5 mm for coarse aggregates, 2 mm for the fine asphalt matrix, and 0.5 mm for buried strain sensors. A total of approximately 15 million tetrahedral elements were generated. Therefore, the computation was performed on a high-performance server equipped with 64 cores, 128 threads, and 1 TB of memory. To reduce memory requirements while maintaining the necessary computational accuracy, the mesh size was optimized to the maximum allowable value.

### 4.3. Material Properties

The mechanical behavior of coarse aggregates in asphalt mixtures is relatively stable and can be effectively represented by a linear elastic model. In this study, the elastic modulus and Poisson’s ratio of the coarse aggregates were set to 50 GPa and 0.2, respectively. The buried strain sensor is also modeled as a linear elastic material. Considering that the encapsulation and flanges of the buried strain sensor are typically made of metallic materials, the elastic modulus and Poisson’s ratio are assigned as 200 GPa and 0.3, respectively. Due to the viscoelasticity of the asphalt binder, asphalt mixtures exhibit strong temperature sensitivity. Therefore, at the mesoscale, it is preferable to characterize the fine aggregate matrix using a viscoelastic model. However, incorporating viscoelasticity at this scale significantly increases the degrees of freedom of the element nodes, especially with highly refined meshes, resulting in excessive computational costs.

**Figure 6:**
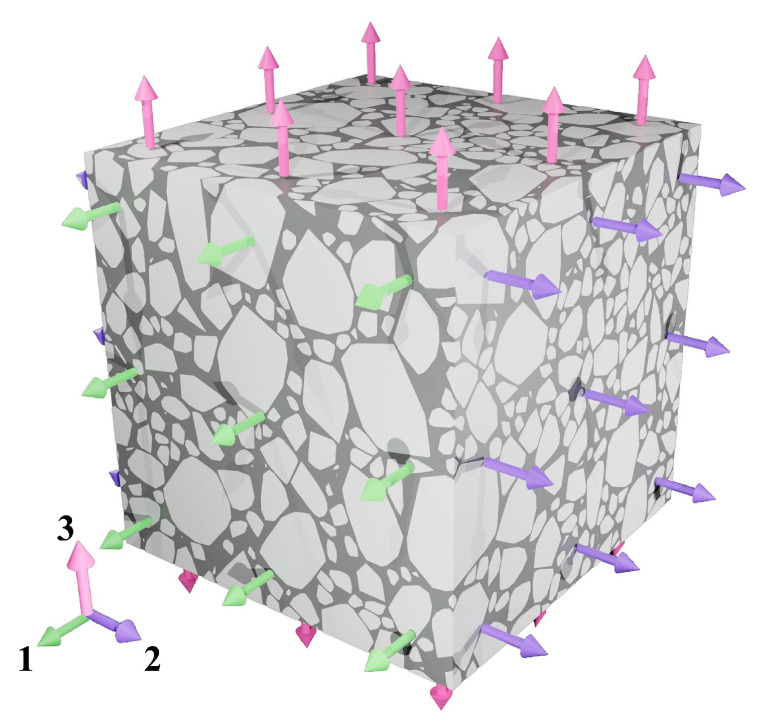
Representative volume element of asphalt mixture.

**Figure 7:**
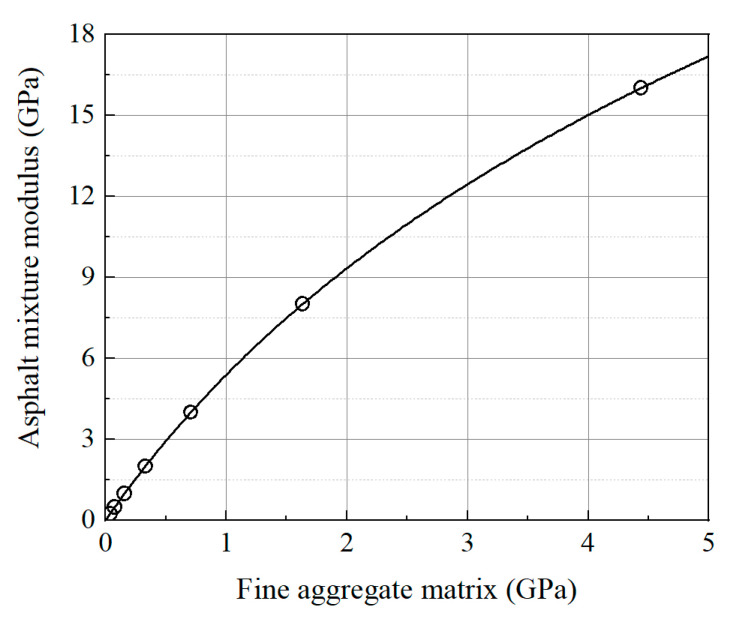
Modulus correspondence between fine aggregate matrix and asphalt mixtures.

To reduce computational demands while maintaining acceptable accuracy, this study employs an equivalent mechanical approach to approximate the viscoelastic response of the asphalt mixture. The viscoelastic behavior of asphalt mixtures under varying temperatures and loading frequencies is discretized into a series of instantaneous elastic problems using composite material homogenization methods, which is a commonly used approach to reduce computational complexity [39,40]. Specifically, the fine aggregate matrix was modeled as a linear elastic material, and the overall mechanical response of the asphalt mixture was calibrated by adjusting the fine aggregate matrix properties through composite homogenization methods. As illustrated in Figure 6, uniaxial loading is applied along three mutually perpendicular orthogonal directions on representative volume elements of appropriate size. The effective modulus of the asphalt mixture can be determined using Equation (1). The dynamic modulus of the asphalt mixture generally ranges from 0.25 GPa to 16 GPa, depending on temperature and loading frequency. To simulate the behavior of the asphalt mixture under various conditions, eight representative moduli (0.25 GPa, 0.5 GPa, 1 GPa, 2 GPa, 4 GPa, 8 GPa, and 16 GPa) were selected for discrete simulations. Therefore, the modulus of fine aggregate matrix was incremented from 0.01 GPa to 5 GPa in steps of 0.01 GPa for parameter scanning. As shown in Figure 7, the equivalent modulus of the asphalt mixture exhibits a monotonically increasing relationship with the modulus of fine aggregate matrix. Specifically, when the asphalt mixture modulus is 0.25 GPa, 0.5 GPa, 1 GPa, 2 GPa, 4 GPa, 8 GPa, and 16 GPa, the modulus of fine aggregate matrix is 0.039 GPa, 0.078 GPa, 0.160 GPa, 0.331 GPa, 0.708 GPa, 1.634 GPa, and 4.439 GPa, respectively.

(1) $$E=\frac{E_{1}+E_{2}+E_{3}}{3},E_{i}=\frac{L}{\Delta L_{i}}\frac{1}{V}\int \sigma_{ii}dV$$

where $E_{i}$ denotes the effective modulus of the representative volume element in the i-th direction, where L and ΔL represent the original length and elongation of the representative volume element, respectively. V is the volume of the representative volume element, and $\sigma_{ii}$ refers to the normal stress in the i-th direction.

### 4.4. Boundary Conditions

Considering that buried strain sensors are primarily used to capture tensile and compressive deformations, this study simulated uniaxial loading conditions. Displacement boundary conditions were applied at both ends of the asphalt mixture specimen. Within the linear elastic framework, the magnitude of the applied load affects only the amplitude of the resulting strain field, not its spatial distribution. Therefore, to obtain a representative strain pattern, the prescribed displacement was defined to induce a unit strain, set as 1 με in this study.

## 5. Measurement Performance Improve of Buried Strain Sensor

### 5.1. Deformation Compatibility of Buried Strain Sensor

**Figure 8:**
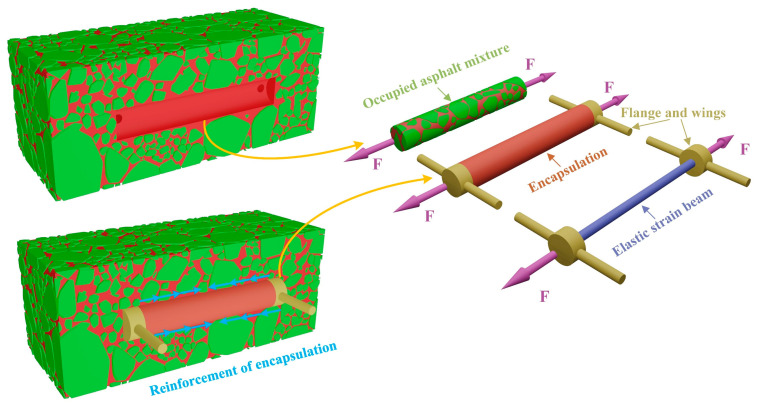
Deformation compatibility between the buried strain sensor and the asphalt mixture.

The deformation compatibility between the buried strain sensor and the asphalt mixture is important for accurately measuring internal strain. This study attributes the deformation incompatibility primarily to the mismatch in deformation stiffness between the buried strain sensor and the asphalt mixture. As illustrated in Figure 8, this mismatch originates from two key factors. First, the equivalent modulus of the buried strain sensor differs from that of the replaced asphalt mixture. This discrepancy varies with the temperature-dependent changes in the modulus of the asphalt mixture. Second, interfacial incompatibility may arise between the buried strain sensor and the asphalt mixture. The encapsulated sensor region acts as a locally stiffened zone, introducing interfacial constraints that limit the deformation of the surrounding asphalt, thereby further reducing compatibility. To gain deeper insights into the above issue, this study independently investigates two key parameters of the buried strain sensor: the equivalent modulus, which influences its deformation behavior, and the encapsulation modulus, which governs its mechanical interaction with the surrounding asphalt mixture.

#### 5.1.1. Effect of Sensor Equivalent Modulus

The equivalent modulus $E_{e}$ of the buried strain sensor is governed by the internal elastic strain beam. Therefore, the equivalent modulus is calculated proportionally based on the ratio of the sectional area $E_{b}$ of the elastic beam to that $A_{s}$ of the entire sensor, as expressed in Equation (2). Therefore, the equivalent modulus of the buried strain sensor in the numerical simulation can be adjusted by modifying the modulus assigned to the internal beam. The modulus of the asphalt mixture is discretized into the following values: 0.25 GPa, 0.5 GPa, 1 GPa, 2 GPa, 4 GPa, 8 GPa, and 16 GPa. The equivalent modulus of the embedded strain sensor is likewise discretized using the same set of values. Consequently, the modulus of the elastic beam is determined to be 2.8 GPa, 5.6 GPa, 11.1 GPa, 22.2 GPa, 44.4 GPa, 88.8 GPa, and 177.8 GPa. The Poisson’s ratio of the elastic beam was consistently set to 0.25. The deformation compatibility and strain measurement accuracy are evaluated for all combinations of these modulus values.

(2) $$E_{e}=\frac{A_{b}E_{b}}{A_{s}}$$

**Figure 9:**
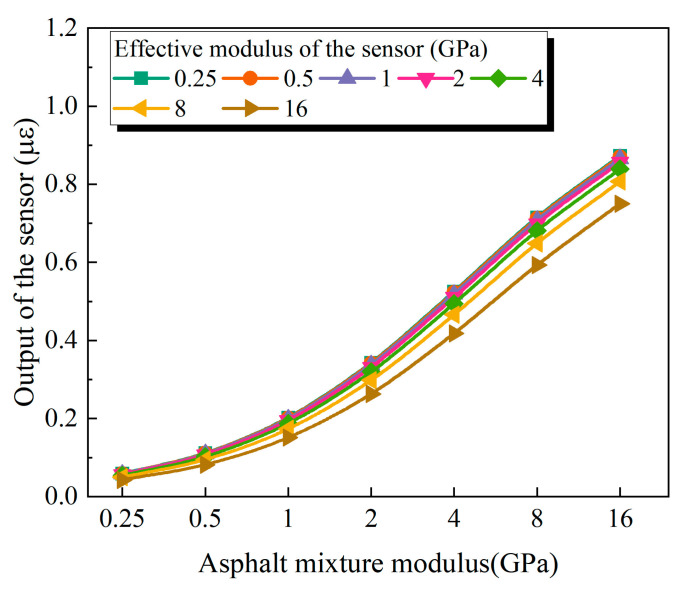
Effect of sensor equivalent modulus.

Figure 9 presents the simulation results for all combinations of sensor and asphalt mixture moduli. When the modulus of the asphalt mixture is fixed, the output strain measured by the buried strain sensor gradually decreases as the sensor modulus increases. It is noteworthy that in all cases, even when the modulus of the sensor is lower than that of the surrounding material, the measured strain remains lower than the actual deformation of the asphalt mixture (1 με). This discrepancy is primarily attributed to the interfacial constraint introduced by the encapsulation layer, which restricts the deformation of the sensor. This reason will be analyzed in detail in the following sections. Moreover, as the modulus of the mixture decreases, the deviation between the sensor output and the actual strain increases. This is primarily because the deformation stiffness of asphalt mixtures typically decreases with increasing temperature. In general, the simulation results presented in this study are consistent with the experimental trends previously reported.

#### 5.1.2. Effect of Sensor Encapsulation Modulus

**Figure 10:**
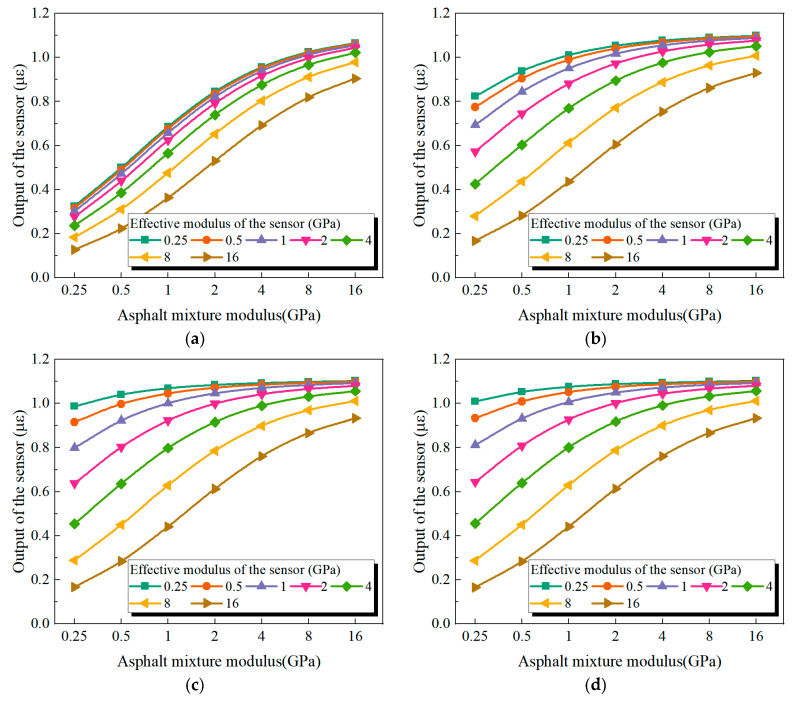
Effect of encapsulation modulus: (**a**) 20 GPa, (**b**) 2 GPa, (**c**) 0.2 GPa, (**d**) 0.02 GPa.

To research the impact of encapsulation modulus on the measurement performance of buried strain sensors, the modulus of the encapsulation material was gradually reduced from 200 GPa to 20, 2, 0.2, and 0.02 GPa. A lower encapsulation modulus weakens the stiffening effect imposed on the surrounding asphalt mixture. Figure 10 presents the sensor responses under different encapsulation modulus conditions. As the encapsulation modulus decreases, the reduction in reinforcement effect is most pronounced for sensors with lower modulus. In contrast, sensors with higher moduli (e.g., 16 GPa) exhibit minimal changes in output under different encapsulation moduli. This trend is primarily attributed to the higher strain sensitivity of low-modulus sensors, which makes them more susceptible to the constraints imposed by the encapsulation. When the encapsulation modulus is sufficiently low (e.g., 0.02 GPa) and the sensor modulus matches that of the asphalt mixture (i.e., 0.25 GPa, 0.5 GPa, 1 GPa, 2 GPa, 4 GPa, 8 GPa, and 16 GPa), the corresponding sensor outputs are 1.0 με, 1.0 με, 1.0 με, 1.0 με, 0.99 με, 0.97 με, and 0.93 με, respectively. These values are close to the actual deformation of the mixture (1 με). Nevertheless, as the sensor modulus increases, the measured strain gradually underestimates the true deformation. This discrepancy may be attributed to the effect of the sensor flange, whose limited thickness induces a non-uniform displacement field along the thickness direction, thereby affecting the overall sensor response. Therefore, minimizing the flange thickness of the buried strain sensor is recommended to improve measurement accuracy.

Moreover, when the reinforcement constraint imposed by the encapsulation is effectively minimized (for example, by setting the encapsulation modulus to 0.25 GPa), low-modulus sensors placed in high-modulus asphalt mixtures tend to overestimate the actual strain. For example, when the sensor modulus is 0.25 GPa and the asphalt mixture modulus is 16 GPa, the sensor output reaches 1.10 με, corresponding to a relative error of 10%. Conversely, high-modulus sensors placed in low-modulus asphalt mixtures significantly underestimate the strain. For example, when the sensor modulus is 16 GPa and the asphalt mixture modulus is 0.25 GPa, the sensor output drops to 0.17, resulting in a relative error as high as 83%. These results suggest that low-modulus sensors consistently yield smaller measurement errors across the entire range of asphalt mixture stiffness, irrespective of modulus matching. Therefore, the use of low-modulus buried strain sensors is recommended for the strain monitoring of asphalt mixtures at different temperature conditions, as they improve the accuracy of the measurements and reduce the errors caused by modulus mismatch.

### 5.2. Measurement Stability of Buried Strain Sensor

**Figure 11:**
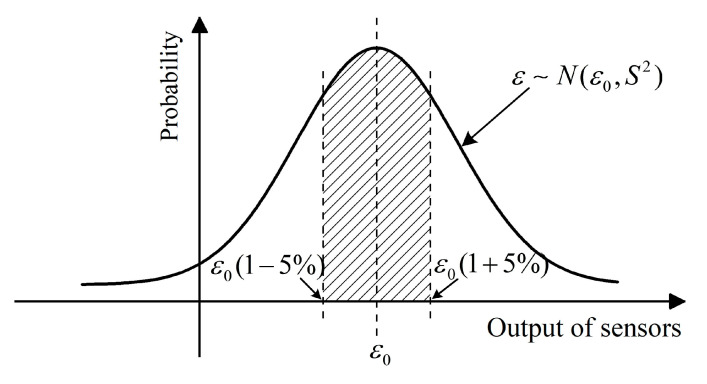
Evaluation of measurement stability of buried strain sensors.

The low-modulus buried strain sensor and free from encapsulation-induced reinforcement effects, has been demonstrated to provide the highest measurement accuracy for asphalt mixtures under varying temperatures and loading frequencies. To further investigate how the heterogeneous meso-structure of asphalt mixtures affects the measurement stability of such sensors, a large number of randomly generated meso-scale asphalt mixture models incorporating low-modulus buried strain sensors were generated. Given the intrinsic randomness of the mixture, the buried strain sensor outputs are assumed to follow a normal distribution $N(\varepsilon_{0},\;S^{2})$, where $\varepsilon_{0}$ denotes the expected strain value and S represents the standard deviation. A 5% relative error margin was adopted as the threshold for evaluating measurement stability. As illustrated in Figure 11, a schematic diagram is provided to estimate the probability that the sensor outputs fall within ±5% of the target value. To ensure a reliable statistical analysis of output variability, simulations and analyses were conducted on 50 parallel meso-structure samples.

#### 5.2.1. Effect of Asphalt Mixture Modulus

This study systematically evaluates the measurement stability of buried strain sensors in mixtures under various temperature conditions and loading frequencies. The mechanical behavior of the asphalt mixture under different temperature and loading environments is characterized by variations in modulus. To eliminate the stiffening effect caused by encapsulation, a low-modulus buried strain sensor is employed, with an equivalent modulus of 0.25 GPa and an encapsulation modulus of 0.02 GPa. The modulus of the asphalt mixture varies from 0.25 GPa to 16 GPa, simulating various mechanical conditions associated with changes in temperature and loading frequency. The modulus of the asphalt mixture ranges from 0.25 GPa to 16 GPa, simulating a wide spectrum of mechanical conditions associated with changes in temperature and loading rate.

**Figure 12:**
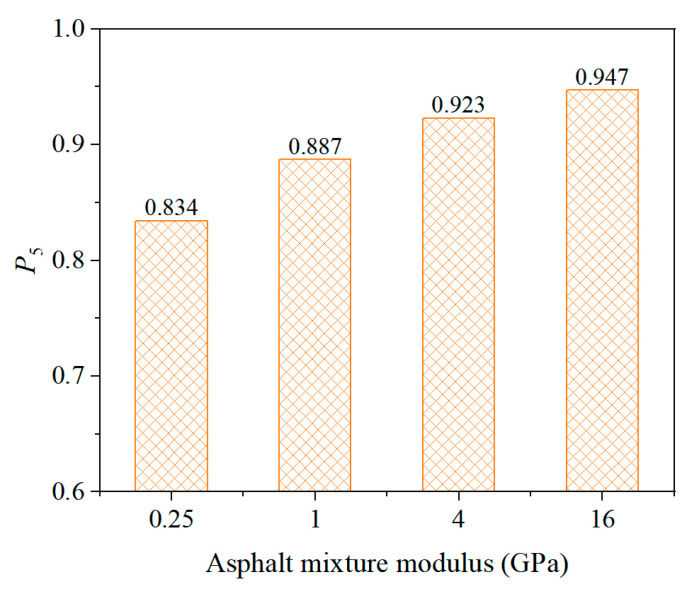
Effect of asphalt mixture modulus.

Figure 12 presents the simulation results. It can be observed that the $P_{5}$ value of the buried strain sensor increases with the modulus of the mixture, indicating improved measurement stability. The heterogeneity of asphalt mixtures primarily arises from the modulus mismatch between the fine aggregate matrix and aggregate particles. In general, the modulus of the aggregates remains constant, while the modulus of the fine aggregate matrix varies with temperature and loading frequency, thereby affecting the overall modulus of the mixture. Lower temperatures or higher loading frequencies result in a higher modulus of the fine aggregate matrix. It is noteworthy that the modulus of the aggregates is significantly higher than that of the fine aggregate matrix. Therefore, an increase in the fine aggregate matrix modulus can reduce the modulus mismatch between the two phases, enhancing the homogeneity of the asphalt mixture. As a result, a higher modulus of the asphalt mixture indicates better homogeneity, which contributes to improved measurement stability of the buried strain sensors.

#### 5.2.2. Effect of Nominal Maximum Particle Size

The gradation of asphalt mixtures, especially NMAS, has a significant effect on the homogeneity of the internal strain field. Due to the inherent heterogeneity of asphalt mixtures, it is generally recommended that the asphalt layer thickness be at least three times the NMAS to mitigate variations caused by material non-uniformity. This study analyzed asphalt mixtures of types AC-13, AC-16, AC-20, and AC-25, with NMAS of 13.2 mm, 16 mm, 19 mm, and 26.5 mm, respectively. Since heterogeneity is most pronounced under low modulus conditions, the modulus of the asphalt mixtures was set to 0.25 GPa for this analysis.

**Figure 13:**
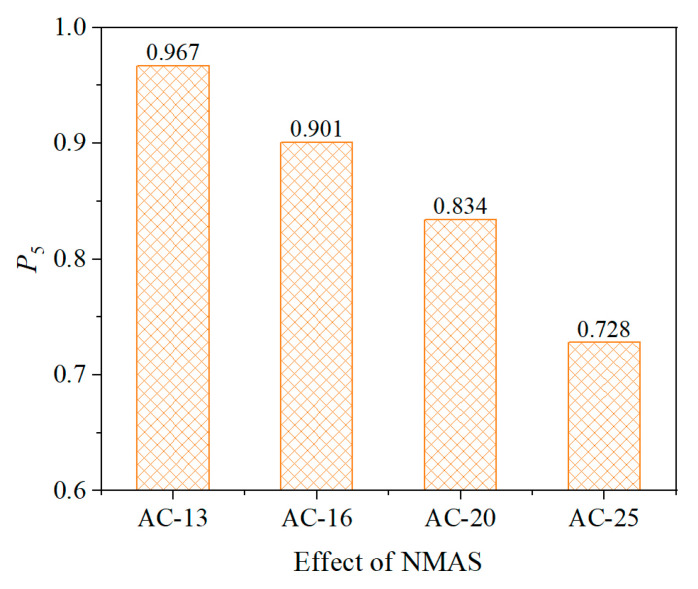
Effect of NMAS.

Figure 13 presents the simulation results. It can be observed that the measurement stability of the buried strain sensor decreases with increasing NMAS of the asphalt mixture. Therefore, when the NMAS of the asphalt mixture is relatively large, the influence of NMAS on sensor stability must be carefully considered, especially under high-temperature conditions. Accordingly, an asphalt mixture with a modulus of 0.25 GPa and an AC-25 gradation was selected for the subsequent sensor parameter investigation. Therefore, when the NMAS of the asphalt mixture is large, the impact of NMAS on sensor stability must be carefully considered, especially under high-temperature conditions. Considering the most unfavorable condition for the uniformity of the internal strain field in the asphalt mixture, the asphalt mixture with a modulus of 0.25 GPa and an AC-25 gradation was selected for the subsequent sensor parameter study.

#### 5.2.3. Effect of Flange Wing

**Figure 14:**
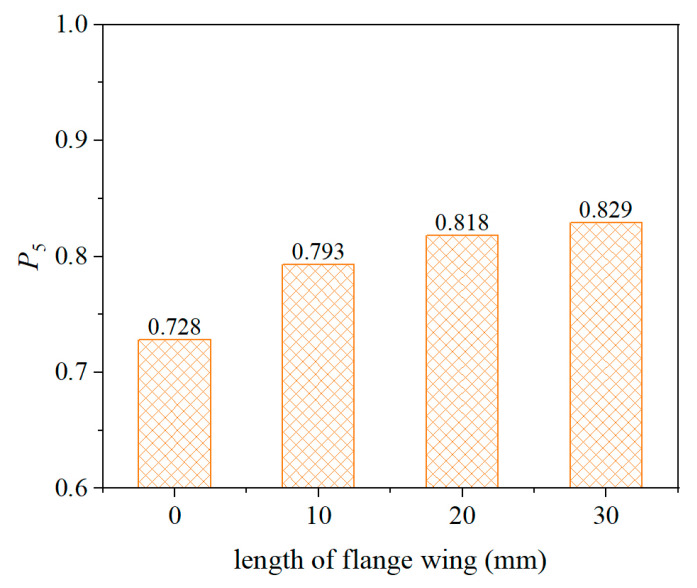
Effect of flange wing.

The addition of wings on the flange may also help improve the measurement stability of buried strain sensors. Therefore, the influence of wing length on sensor measurement stability was investigated by introducing wings of 10 mm, 20 mm, and 30 mm in length on the sensor flange. To ensure that the buried strain sensor output accurately reflects the real strain of the mixture, the equivalent modulus of the buried strain sensor was set to 0.25 GPa, and the modulus of the encapsulated was set to 0.02 GPa. As shown in Figure 14, the probability of sensor outputs falling within ±5% of the mean value for different wing lengths is presented. The results indicate that adding wings and increasing their length can enhance the measurement stability of the sensor. However, when the wing length increases from 20 mm to 30 mm, the improvement becomes negligible, indicating that the stabilizing effect of the wings is limited.

#### 5.2.4. Effect of Sensor Gauge Length

The gauge length of the buried strain sensor has a significant impact on its measurement stability. In this study, the gauge length is defined as the distance between the sensor flanges. Increasing the gauge length enables the sensor to capture the average strain over a larger area of the composite material, thereby enhancing measurement stability. However, existing related studies are very limited and lack quantitative evaluation. To address this issue, the present study analyzes buried strain sensors with gauge lengths of 40 mm, 60 mm, 80 mm, and 100 mm. The sensors feature a low modulus and unreinforced package design. The asphalt mixture exhibits a relatively low overall modulus of 0.25 GPa.

**Figure 15:**
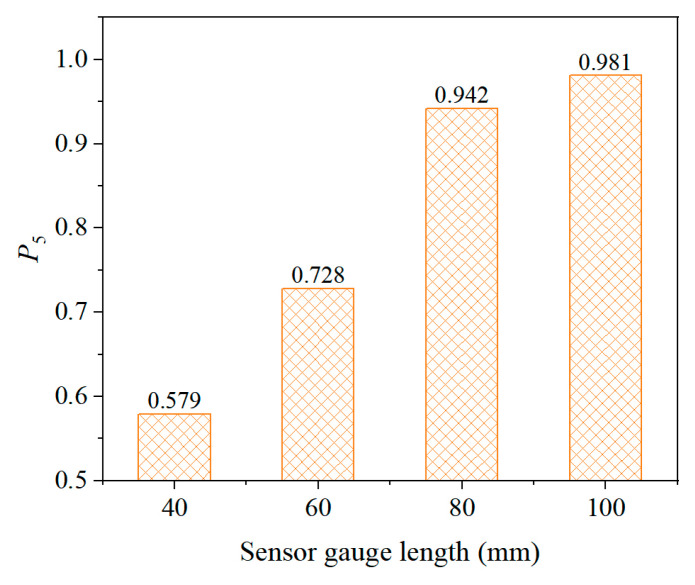
Effect of sensor gauge length.

As shown in Figure 15, the sensing stability of the buried strain sensor significantly improves with increasing gauge length. When the gauge length is 40 mm, the probability of the measured value falling within ±5% of the mean is 0.579. This probability sharply increases to 0.728 at a gauge length of 60 mm, and further rises to 0.942 at 80 mm, indicating excellent measurement stability. Therefore, increasing the gauge length of the buried strain sensor is one of the most effective ways to enhance sensor stability. Notably, the gauge length of the sensor is approximately three times the NMAS of the asphalt mixture, which closely aligns with the engineering practice that the asphalt layer thickness should not be less than three times the NMAS. This consistency confirms the reliability of the simulation results.

## 6. Conclusions

This study systematically investigates the performance and enhancement of buried strain sensors in asphalt mixtures using mesoscale finite element analysis. The main conclusions are as follows:

(1) The measurement performance of buried strain sensors in asphalt mixtures is influenced by the viscoelastic behavior of the material. This is mainly because, as temperature increases, the modulus of the asphalt mixture decreases, resulting in a mismatch between the modulus of the mixture and that of the buried strain sensor.

(2) The heterogeneity of the asphalt mixture affects the measurement stability of the buried strain sensor. The greater the overall modulus of the asphalt mixture, the more uniform the internal strain distribution. Additionally, the larger the nominal maximum aggregate size, the poorer the uniformity of the strain field.

(3) To ensure high measurement accuracy, the modulus of the buried strain sensor should be identical to that of the asphalt mixture, and the encapsulation should not introduce a stiffening effect. Considering the dynamic modulus range of the asphalt mixture, it is recommended to adopt the lower bound, such as 0.25 GPa, as the equivalent modulus of the buried sensor to ensure that the maximum measurement error remains within 10%. The encapsulation can consider using low-modulus flexible materials to eliminate the reinforcing effect.

(4) The flange wing and gauge length of the buried strain sensor affects its measurement stability in asphalt mixtures. As the length of flange wing or gauge length increases, the measurement stability of the sensor increases. However, while the flange wing contributes to the stability to some extent, the effect of increasing the gauge length is much more pronounced. It is recommended that the gauge length of the buried sensor be at least three times greater than the NMAS of the asphalt mixture.

This study established a mesoscale finite element framework to investigate the strain transfer behavior of embedded sensors in asphalt mixtures, providing theoretical guidance for sensor design optimization. While the findings reveal important insights into the effects of modulus matching, encapsulation stiffness, and gauge length, experimental validation has not yet been conducted. In future work, laboratory testing will be carried out to verify the simulation results and refine the model accordingly.

## Data Availability

Data are contained within the article.
